# A phase I/II trial to evaluate the safety, feasibility and activity of salvage therapy consisting of the mTOR inhibitor Temsirolimus added to standard therapy of Rituximab and DHAP for the treatment of patients with relapsed or refractory diffuse large cell B-Cell lymphoma – the STORM trial

**DOI:** 10.1186/1471-2407-13-308

**Published:** 2013-06-25

**Authors:** Mathias Witzens-Harig, Marie Luise Memmer, Martin Dreyling, Georg Hess

**Affiliations:** 1 Department of Internal Medicine V, University of Heidelberg, INF 410, 69120, Heidelberg, Germany; 2 Department of Internal Medicine III, University of Munich (LMU), Marchioninistr. 15, Munich 81377, Germany; 3 Department of Hematology, Oncology, and Pneumology, University of Mainz, Langenbeckstr 1, 55131, Mainz, Germany

## Abstract

**Background:**

The current standard treatment of patients with relapsed or refractory diffuse large cell B-Cell lymphoma (DLBCL) primarily consists of intensified salvage therapy and, if the disease is chemo-sensitive, high dose therapy followed with autologous stem cell transplantation. In the rituximab era however, this treatment approach has shown only limited benefit. In particular, patients relapsing after rituximab-containing primary treatment have an adverse prognosis, especially if this occurs within the first year after therapy or if the disease is primarily refractory. Therefore there is an ultimate need for improved salvage treatment approaches.

**Methods/design:**

The STORM study is a prospective, multicentre phase I/II study to evaluate the safety, feasibility and activity of salvage therapy consisting of the mTOR inhibitor temsirolimus added to the standard therapy rituximab and DHAP for the treatment of patients with relapsed or refractory DLBCL. The primary objective of the phase I of the trial is to establish the maximum tolerated dose (MTD) of temsirolimus in combination with rituximab and DHAP. The secondary objective is to demonstrate that stem cells can be mobilized during this regimen in patients scheduled to proceed to high dose therapy. In phase II, the previously established maximum tolerated dose of temsirolimus will be used. The primary objective is to evaluate the overall response rate (ORR) in patients with relapsed DLBCL. The secondary objective is to evaluate progression free survival (PFS), overall survival (OS) and toxicity. The study will be accompanied by an analysis of lymphoma subtypes determined by gene expression analysis (GEP).

**Discussion:**

The STORM trial evaluates the safety, feasibility and activity of salvage therapy consisting of the mTOR inhibitor temsirolimus added to standard therapy of rituximab and DHAP for the treatment of patients with relapsed or refractory DLBCL. It also might identify predictive markers for this treatment modality.

**Trial Registration:**

ClinicalTrials.gov NCT01653067

## Background

Non-Hodgkin’s Lymphomas are the fifth most common tumor type worldwide, and their incidence is still increasing [1]. Although in recent years advances in tumor therapy and supportive care have improved overall survival, a large proportion of patients will ultimately die of their disease. The prognosis of diffuse large B-cell lymphoma (DLBCL) has improved with the advent of the monoclonal antibody rituximab. However, there is increasing evidence that treatment of patients with relapsed and refractory disease remains challenging. The current standard treatment of patients with relapsed or refractory DLBCL primarily consists of intensified salvage therapy using widely accepted regimens like R-DHAP or R-ICE. For chemo-sensitive disease high dose therapy followed with either autologous [2] or, in selected cases, allogeneic transplantation is applied. In the rituximab era however, high dose therapy and autologous transplantation have only been of limited benefit in relapsed and refractory disease, and allogeneic transplantation is limited to a selected small subset of patients. The dismal prognosis was recently underlined by the interim analysis of the CORAL trial, in which patients with relapsed DLBCL were randomized to either receive salvage R-DHAP or R-ICE: it was demonstrated that patients relapsing after rituximab-containing primary treatment had an adverse prognosis, especially if this occured within the first year after therapy or if the disease was primarily refractory. Even with this intensive treatment in this patient subset only 10-15% of patients achieve long-term survival [3].

Recently, the specific mTOR inhibitor temsirolimus has shown to be clinical active in relapsed mantle cell lymphoma in a large multicenter phase III trial, which included patients with up to 7 prior lines of therapy. In this poor-risk population, the ORR was 22% using a regimen consisting of 175 mg temsirolimus for 3 weeks given weekly, followed by 75 mg/weekly or 25 mg/weekly until tumor progression or unacceptable toxicity occured. During the later therapy phase the average dose was 52 mg/week. The most prominent side effect in this trial was thrombocytopenia. PFS, which was the primary endpoint of this trial, was significantly superior using this regimen, in comparison to a standard treatment arm, which consisted of a variety of commonly accepted single agents. Interestingly, the superiority of temsirolimus appeared to be accentuated in patients with a lower number of pre-treatments [4]. In another trial, the combination of rituximab and even low dosis of temsirolimus resulted in impressive response rates in relapsed and refractory mantle cell lymphoma [5].

Furthermore, a recently presented phase II trial by Smith and colleagues demonstrated single agent activity of temsirolimus in DLBCL and follicular lymphoma by achieving a ORR of 56% in relapsed patients. Especially a single agent activity of 28% in relapsed aggressive lymphoma is promising and merits further evaluation [6].

It seems therefore a logical consequence to incorporate temsirolimus into earlier treatment lines or to combine it with other therapies. Accordingly, a combination of temsirolimus with bendamustine and rituximab achieved a response in all patients evaluable with relapsed mantle cell and follicular lymphoma [7]. Of note, in recent in vitro experiments, additive action of temsirolimus, dexamethasone, cytarabine and platinum could be demonstrated [8]. Building on to this, the STORM trial combines temsirolimus with a well-established salvage treatment protocol (R-DHAP) with a known safety profile for the treatment of patients with refractory or relapsed DLBCL. The aim of this trial is to determine the safety, feasibility and clinical activity of the proposed regimen.

## Methods/design

### Trial organization

The STORM trial has been designed by the Trial Centre of the Department of Hematology and Oncology of the University of Heidelberg in cooperation with the Department of Hematology and Oncology of the University of Mainz and the other participating centres. The trial is an investigator initiated trial, and is sponsored by the University Hospital of Heidelberg. The trial is coordinated by the Department of Hematology and Oncology of the University of Heidelberg, which is responsible for the overall trial management, trial registration (ClinicalTrials.gov Identifier NCT01653067) and the scientific program of all trial-related meetings. Database management, quality assurance, monitoring and reporting is performed by the Interdisciplinary Centre for Clinical Trials (IZKS) at the University of Mainz.

A total of 9 German centres participate in this trial. The centres are (listed alphabetically): University Hospital Charité, Berlin; University Hospital Erlangen; University Hospital Frankfurt, University Hospital Freiburg; University Hospital Heidelberg; University Hospital Mainz; University Hospital Munich LMU; University Hospital Munich TU and University Hospital Ulm.

### On-site monitoring

During recruitment on-site monitoring is performed following good clinical practice (GCP) guidelines. The data management will be performed by the Interdisciplinary Centre for Clinical Trials (IZKS) at the University of Mainz.

### Ethics, informed consent and safety

The final protocol was jointly approved by the central ethics committee of this trial at the University of Heidelberg, Medical School (AFmu-017/2012, http://www.klinikum.uni-heidelberg.de) and by the ethics committees of all participating centres. This study complies with the Helsinki Declaration in its most recent German version, the Medical Association's professional code of conduct, the principles of Good Clinical Practice (GCP) guidelines and the Federal Data Protection Act. The trial will also be carried out in keeping with local legal and regulatory requirements. The medical secrecy and the Federal Data Protection Act will be followed.

Informed consent is obtained from each patient in oral and written form before inclusion in the trial and after the nature, scope, and possible consequences of the trial have been explained by a physician. The investigator will refrain from any measures specifically required only for the clinical trial until valid consent has been obtained.

### Study design and objectives

The STORM study is a prospective phase I/II study to evaluate the safety, feasibility and activity of a salvage therapy consisting of the mTOR inhibitor temsirolimus added to standard therapy of rituximab and DHAP for the treatment of patients with relapsed or refractory DLBCL. The STORM-trial consists of two phases.

Phase I is a dose-escalation study of temsirolimus. The primary objective is to establish the maximum tolerated dose (MTD) of temsirolimus in combination with rituximab and DHAP. The secondary objective is to demonstrate that stem cells can be mobilized during this regimen in patients scheduled to proceed to high dose therapy.

In phase II, the previously established maximum tolerated dose of temsirolimus will be used. The primary objective is to evaluate the overall response rate (ORR) in patients with relapsed DLBCL. The secondary objective is to evaluate progression free survival (PFS), overall survival (OS) and toxicity.

### Patient selection

To be included into the STORM trial, patients must be at least 18 years old and have a histologically confirmed diagnosis of DLBCL according to the World Health Organization classification. There must be a documented relapse or progression after at least one prior treatment but a maximum of two prior treatments. Prior treatment must have included at least three cycles of anthracycline-containing chemotherapy (e.g. CHOP-like). The histology has to be confirmed by a reference pathologist. Evaluation of CD20 status is compulsory. At least one measurable tumor mass (>1.5 cm x >1.0 cm), involvement of any organ or bone marrow infiltration must be present. In addition, adequate organ function and a Eastern Cooperative Oncology Group [ECOG] performance Status of less than 3 are essential for inclusion into the trial. Patients are required to use adequate contraception before entry and throughout the study, if appropriate. Naturally, patients must have signed an informed consent document indicating that they understand the purpose of and procedures required for the study and are willing to participate in the study.

Patients with lymphoma other than DLBCL or active central nervous system lymphoma are not eligible. Other exclusion criteria are severe concomitant diseases, active uncontrolled infections including HIV, active hepatitis B or C or other malignant disease (except: adequately treated non-melanoma skin cancer, curatively treated in-situ cancer of the cervix, DCIS of the breast, or other solid tumors curatively treated with no evidence of disease for >5 years). Prior treatment with temsirolimus, known CD20 negativity, disease refractory to DHAP in a prior treatment line, severe psychiatric illness, peripheral neuropathy or neuropathic pain grade 2 or worse, prior autologous or allogeneic stem cell or bone marrow transplantation, concurrent treatment with another investigational agent during the conduct of the trial or known intolerance to sirolimus or derivates, cytarabine, cisplatine or rituximab will prohibit inclusion, as well as pregnancy or breast feeding.

### Statistical design

In phase I of the study, a 6 + 6 standardized design is chosen to establish the MTD of the investigational product (temsirolimus). A maximum of 48 patients can consequently be included into this phase. In phase II of the study, 40 patients will be included, receiving two to four cycles of the full target dose of temsirolimus in combination with R-DHAP, as established in phase I of the trial. The number of cycles will depend on the evaluation of activity and toxicity of the regimen by the investigator in each individual patient. Based on published data on rituximab in combination with DHAP, a response rate of at least 60 to 65% (40% CR and CRu) can be expected. The study will be terminated if the number of non-responders exceeds a critical value determined by Wald´s Sequential probability ratio test. In both phases of this trial explorative statistics are used to calculate remission and response rates. Median progression free survival, overall survival etc. are calculated by the Kaplan and Meier method. Adverse events will be classified according to MedDRA terminology. The frequency of adverse events will be calculated, and there will be further analyses to determine their seriousness, intensity, duration, relationship to trial treatment, actions taken and clinical outcome. Patients with and without consolidating high dose therapy and autologous stem cell transplantation will be analysed separately.

### Work up

Patients with histologically documented diagnosis of DLBCL receive a complete work-up which includes cervical, thoracic, abdominal and pelvic CT scans, bone marrow histology and extensive laboratory testing. The IPI risk score will be calculated for each patient. All in- and exclusion criteria are evaluated. If patients meet all inclusion criteria, they will be informed about the study and all associated risks and benefits, and their written consent is sought. If patients decline treatment within the STORM trial, an adequate alternative treatment regimen is offered.

### Treatment

This is a multicenter, open label, single arm, phase I/II study. Placebo will not be used within this trial. In phase I, the dose escalation phase of this trial, 6 patients will be included in each dose level. There will be four cohorts, administering up to a maximum of four cycles with 25 mg, 50 mg, 75 mg or 100 mg temsirolimus on day 1 and 8 in combination with rituximab (375 mg/m^2^ day 2) and DHAP (dexamethasone 40 mg day 3–6, cisplatin 100 mg/m^2^ day 3, cytarabine 2 × 2 g/m^2^ day 4). Treatment is repeated on day 22 for up to a maximum of 4 cycles.

**Figure 1:**
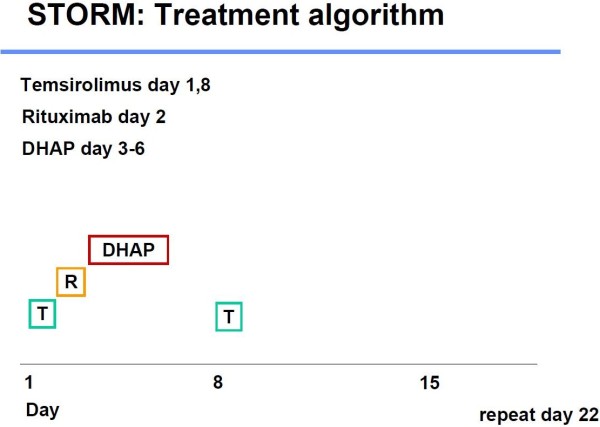
Treatment algorithm of the STORM trial.

After inclusion of six patients, each patient has to receive at least one complete cycle without experiencing any dose limiting toxicity, until the enrolment into the next cohort can be initiated. In phase II of the trial 40 patients will be included to receive the previously established full target dose. Special attention in phase I and phase II of the study is brought to monitoring of adverse events. Stem cell mobilization and subsequent high dose therapy and autologous stem cell transplantation can be performed in eligible patients (Figure 1).

### Safety and discontinuation of treatment

Toxicities are classified by grade, type, duration, onset, and relationship to study treatment using the NCI Common Toxicity Criteria (CTC). Dose limiting toxicities of STORM in the context of the trial treatment are defined as any CTCAE grade V toxicity that are potentially related to the trial treatment, any hematological toxicity not recovering to at least NCI CTCAE grade II after 28 days after start of the last STORM-cycle (except as a consequence of bone marrow insufficiency due to bone marrow infiltration) and any non-hematological toxicity NCI CTCAE grade III/IV not recovering to grade II within 14 days after initial occurrence and potentially related to the trial treatment.

In phase I of the trial 6 patients will be included at each dose level. After inclusion of six patients, each patient has to receive at least one complete cycle without experiencing any dose limiting toxicity (DLT), until the enrolment into the next cohort can be initiated. If one DLT occurs, this is discussed with the data safety monitoring board (DSMB). The DSMB may recommend that six additional patients will be added to the specific dose level. If two DLTs occur in the first six patients of a cohort, six additional patients will be added to the specific dose level and this will be discussed with the DSMB. If three DLTs occur in the 6 + 6 cohort, the DSMB will be informed and will recommend further action. If a fourth DLT appears in the 6 + 6 cohort, the last dose level with three or less than three DLTs will be considered the standard dose for the phase II trial. If four DLTs occur in cohort A, the study will continue with an additional Cohort X with a temsirolimus dose of 15 mg. If the final dose level is achieved without any DLT, there will be no further dose escalation. Additionally, cumulated data of each dose level will be presented to the DSMB prior to proceeding to the next dose level. The 6 + 6 design was chosen to provide a more robust data basis than a 3 + 3 design.

### Trial duration

Patient recruitment is planned to be completed after 24 months. Patients will be monitored for three years after study entry. The total duration of the trial is estimated to be five years. Recruitment will begin in April 2013.

### Assessment of therapeutic efficiency

Assessments including patient history, physical exam, CT of neck, chest and abdomen, bone marrow biopsy and serological tumor markers are scheduled before, during and after treatment and during follow up. Response is evaluated in accordance with the Cheson Criteria [9]:

• Complete response (CR) is the complete disappearance of all detectable evidence of disease on CT, and all disease-related symptoms, and normalization of biochemical abnormalities, and normal bone marrow biopsy (BMB). Previously involved nodes on CT more than 1.5 cm in their greatest axial diameter must regress to less than 1.5 cm, and previously measured nodes of 1.1–1.5 cm must decrease to less than 1 cm.

• CRu (uncertain) corresponds to CR criteria but with a residual mass more than 1.5 cm in greatest axial diameter that has regressed by more than 75%.

• Partial response (PR) is at least 50% reduction in the sum of the product of the greatest diameters (SPD) of the six largest nodes with no increase in the size of other nodes and no new sites of disease. Splenic and hepatic nodules must regress by at least 50% in the SPD.

• Stable disease (SD) is less than a PR but is not progressive disease. Progressive disease (PD) is more than 50% increase in the sum of the product of the greatest diameters of any previously abnormal node, or appearance of any new lesions during or at the end of therapy.

• Relapsed disease (RD) is the appearance of any new lesion or increase in size of more than 50% of previously involved sites or nodes in patients who achieved CR or CRu.

PET-data will be collected if available upon discretion of the individual physician.

### Quality assurance program

Quality assurance, database management, monitoring and reporting is performed by the Interdisciplinary Centre for Clinical Trials (IZKS) at the University of Mainz.

## Discussion

The STORM trial evaluates the safety, feasibility and activity of salvage therapy consisting of the mTOR inhibitor temsirolimus added to standard therapy of rituximab and DHAP for the treatment of patients with relapsed or refractory DLBCL. It also might identify predictive markers for this treatment modality.

## Competing interests

The trial is funded by Pfizer Inc., New York, USA. Funding includes trial organization and monitoring by the IZKS Mainz, the statistical analysis, data management and the supply of the study medication. There is no other funding of the trial. The authors also participate in other scientific trials which are supported by Pfizer Inc., New York, USA.

## Authors’ contribution

MWH, MD and GH planned the study and wrote the manuscript. MLM coordinated and conducted the study. All authors read and approved the final manuscript.

## Pre-publication history

The pre-publication history for this paper can be accessed here:

http://www.biomedcentral.com/1471-2407/13/308/prepub
